# HHV-6 Infection in a 19-Year-Old Liver Transplant Recipient – Much More Than Roseola!

**DOI:** 10.1016/j.idcr.2023.e01863

**Published:** 2023-07-26

**Authors:** Alleyna Dougherty, Nathan DeRon, Leigh Hunter

**Affiliations:** a Burnett School of Medicine, Texas Christian University, Fort Worth, TX, United States; b Department of Internal Medicine, Methodist Dallas Medical Center, Dallas, TX, United States; c Department of Infectious Diseases, Methodist Dallas Medical Center, Dallas, TX, United States

**Keywords:** HHV-6, Human herpesvirus-6, Roseola, Liver transplant, Immunosuppression

## Abstract

Human herpesvirus 6 (HHV-6) infections, most commonly occurring during childhood, are frequently mild and self-limited. However, immunosuppression due to transplantation may cause reactivation of HHV-6 with manifestations ranging from fever and skin exanthem to pneumonitis, hepatitis, encephalitis, and myelitis. Because these infections may be devastating for liver transplant recipients leading to transplant organ fibrosis and failure, it is imperative that internists recognize the manifestations, establish early diagnosis, institute appropriate therapy, and make timely referrals to transplant specialists. We present a case of a 19-year-old liver transplant recipient with HHV-6 viremia, encephalopathy, and hepatitis. The patient’s symptoms improved with ganciclovir and intravenous immunoglobulin treatment, serum HHV-6 copies gradually decreased, and she was discharged with outpatient follow-up. After approximately one month of antiviral therapy, the patient’s viral load was undetectable. Early recognition of HHV-6 viremia, appropriate laboratory assessment, and early institution of therapy is important for internal medicine physicians to decrease morbidity and mortality in liver transplant recipients.

## Introduction

Human herpesvirus-6 (HHV-6) infection commonly occurs during childhood, is frequently mild and self-limited, and results in adult prevalence of > 90%. HHV-6 exists as two related species, HHV-6A and HHV-6B. HHV-6B is predominant in the general population, while HHV-6A is more prevalent in immunocompromised hosts [1]. Immunosuppression due to transplantation may cause reactivation of HHV-6A with clinical manifestations ranging from fever and skin exanthem to pneumonitis, hepatitis, and encephalitis [2]. To that end, we present the case of a 19-year-old female liver transplant recipient with a newly diagnosed HHV-6A infection complicated by acute encephalitis. We also discuss common presentations and management of this disease with emphasis on building clinician education and awareness.

## Case Report

**Fig. 1:**
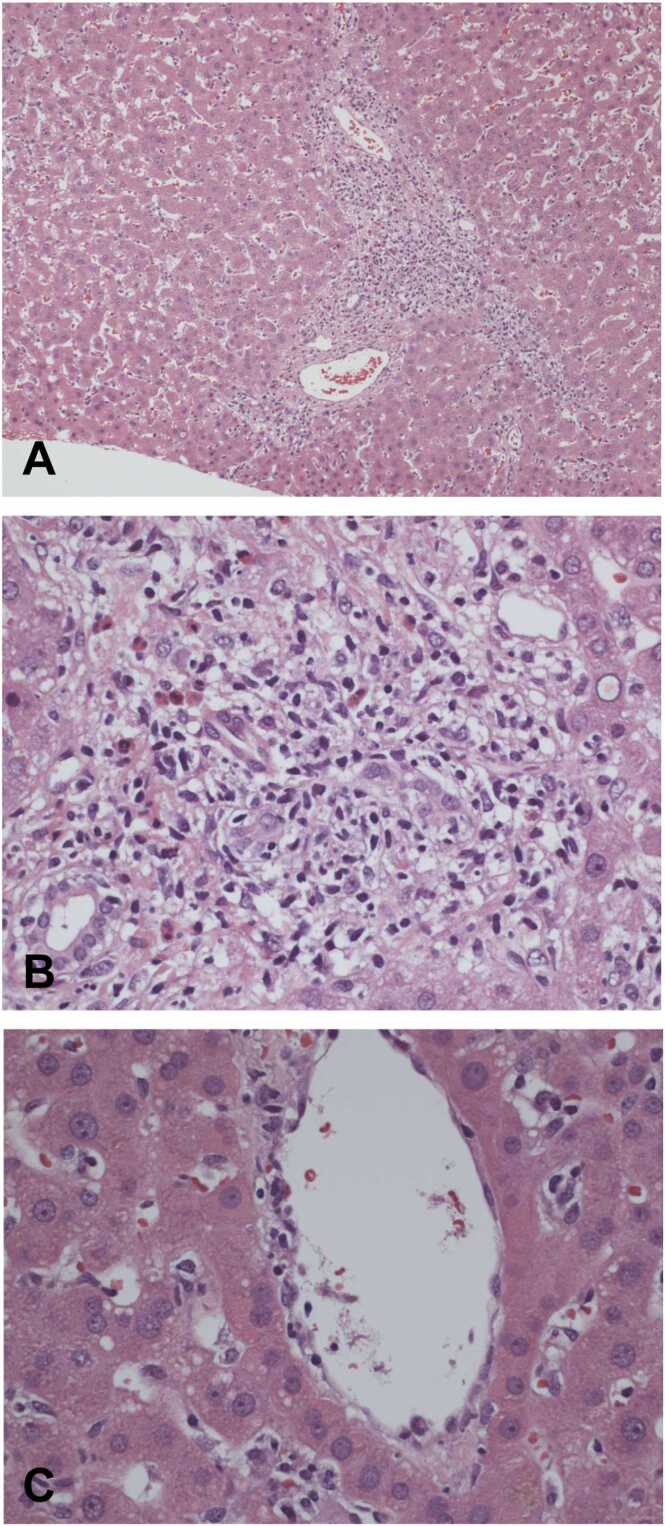
Liver histology showing (A) inflammatory infiltration of portal tract (H&E, x100); (B) portal tract with plasma cells, eosinophils, and lymphocytes with biliary injury (H&E, x40); and (C) central vein endotheliitis (H&E, x40). H&E, hematoxylin and eosin.

A 19-year-old female with a past medical history of acute liver failure secondary to autoimmune hepatitis (status post orthotopic liver transplantation complicated by biliary stricture and recurrent episodes of acute cellular rejection; Fig. 1) presented to the hospital after being found down and unresponsive. Her family reported that the patient was intermittently confused for two to three days prior to admission and complained of a headache during this period. The patient was found down at home with rapid eye movement, left upper extremity tonic-clonic motions, and blood near her nose and mouth. The patient was taken to an outside facility where she experienced a witnessed seizure aborted with lorazepam. During transport to our facility, she was again witnessed to experience another seizure requiring abortive therapy. She was compliant with her immunosuppression regimen: tacrolimus (6 mg twice daily), mycophenolate (1000 mg twice daily), and prednisone (20 mg daily). On examination, the patient was ill-appearing, responsive only to pain, and had sluggish pupils bilaterally. She met sepsis criteria with leukocytosis, tachycardia, and end-organ damage in the form of acute kidney injury and encephalopathy. Computed tomography (CT) images of the head showed areas of hypodensity involving the gray-white matter junction in the bilateral frontal lobes. No new abnormalities were noted on chest radiography, echocardiography, or CT of the abdomen/pelvis. The patient’s initial emergency department evaluation was further complicated by a witnessed tonic-clonic seizure aborted with benzodiazepine therapy.

**Fig. 2:**
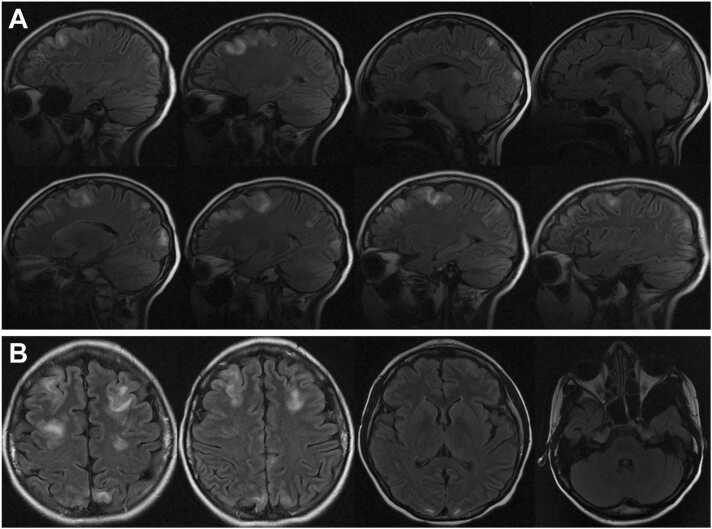
Magnetic resonance imaging T2 fluid attenuated inversion recovery sequence images of the brain. (A) Sagittal view illustrating multifocal areas of hyperintensity consistent with viral encephalitis in both the right (top row) and left (bottom row) brain. (B) Axial view illustrating multifocal areas of hyperintensity consistent with viral encephalitis in the bilateral frontal and occipital lobes with cerebellar sparing.

The patient underwent extensive evaluation, including assessment of serum electrolytes, renal function evaluation, liver enzyme levels, complete blood count, coagulation assays, infectious diseases testing, and further neuroimaging. Electroencephalography showed very low amplitude (4–5 Hz theta) background activity and underlying 1–1.5 Hz generalized delta activity suggestive of moderate to severe generalized cerebral edema. No epileptiform activity was observed. Magnetic resonance imaging (MRI) of the brain showed areas of cortical and subcortical T2 hyperintensity with peripheral restricted diffusion and lesions involving the frontal, parietal, and occipital lobes (Fig. 2). A lumbar puncture with cerebrospinal fluid culture and Gram stain showed no bacterial growth and was negative for West Nile virus, syphilis, herpes simplex viruses 1 and 2, and enterovirus. Additional serologies obtained early in the patient’s hospital course, including JC virus, adenovirus, Epstein-Barr virus, cytomegalovirus, *Histoplasma*, *Aspergillus*, and *Cryptococcus* serologies were negative.

**Fig. 3:**
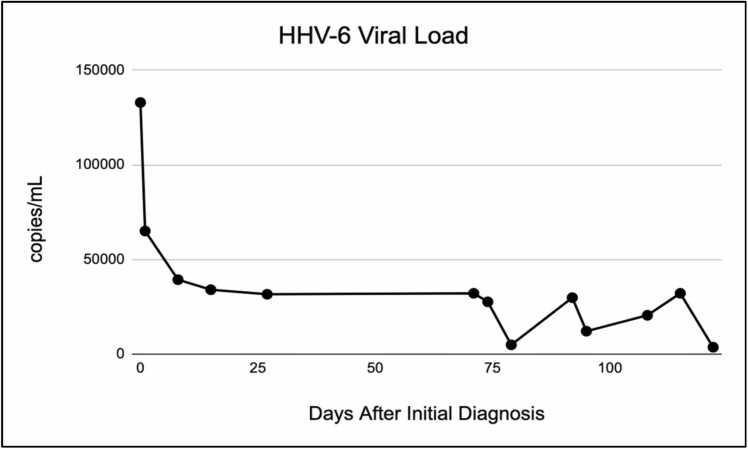
Serum HHV-6 viral load over time. The x-axis indicates the days after admission. The points show the copies of HHV-6 per mL of serum.

Though the patient’s initial neurologic and infectious evaluations left little definitive explanation for her clinical presentation, her HHV-6A serum viral load was 133,000 copies/mL. This was approximately 18 months after initial liver transplant. She was initiated on ganciclovir at a dose of five milligrams per kilogram every 12 h for 14 days before moving to daily dosing. She was also started on intravenous immunoglobulin therapy at a dose of 250 milligrams per kilogram daily for five days. Repeat lumbar puncture was negative for HHV-6A and repeat MRI (status post three days of ganciclovir and intravenous immunoglobulin therapy) showed significant improvement with near complete resolution of the previously demonstrated multifocal subcortical signal abnormalities. The HHV-6A viral load continued to decrease during the remainder of the patient’s hospital stay from 133,000 copies to 34,000 copies at the time of discharge (Fig. 3). Overall, the patient’s clinical status improved, confusion resolved, and she was discharged home in stable condition.

After approximately one month of antiviral therapy, the patient’s HHV-6A viral load was undetectable and antiviral therapy was subsequently discontinued due to concern for bone marrow suppression. The patient was readmitted two months later for recurrent hepatic failure with a concomitant elevated HHV-6A viral load. The patient was restarted on ganciclovir therapy with subsequent adequate viral load suppression. Most recently, her ganciclovir was held due to pancytopenia and type 2 renal tubular acidosis, but she had sustained viral load suppression off antiviral therapy.

## Discussion

A member of the *Herpesviridae* family, HHV-6 is a linear, double-stranded DNA virus 160–162 kb in size. It displays a broad cellular and tissue tropism, including, most efficiently, CD4^+^ T lymphocytes, fibroblasts, natural killer cells, oligodendrocytes, microglia, brain, kidney, liver, and tonsils [3]. HHV-6B is the causal agent in the often self-limiting childhood disease exanthem subitem or roseola infantum. The relationship between HHV-6A and human disease remains poorly understood but is being increasingly recognized in the immunocompromised host. In a small cohort of renal transplant recipients, Okuno et. al. isolated neutralizing antibodies to HHV-6 prior to transplantation, eight of which showed significant increases in HHV-6 antibody titers post-transplantation [4]. More recently, Yoshikawa et. al. observed HHV-6 viremia two to four weeks post-transplantation in bone marrow transplant recipients [5]. However, it is important to remember that HHV-6 can integrate into the cellular genome; therefore, care must be taken to clinically confirm active infection with correlation of symptoms and imaging findings rather than simply relying on serum testing alone.

**Fig. 4:**
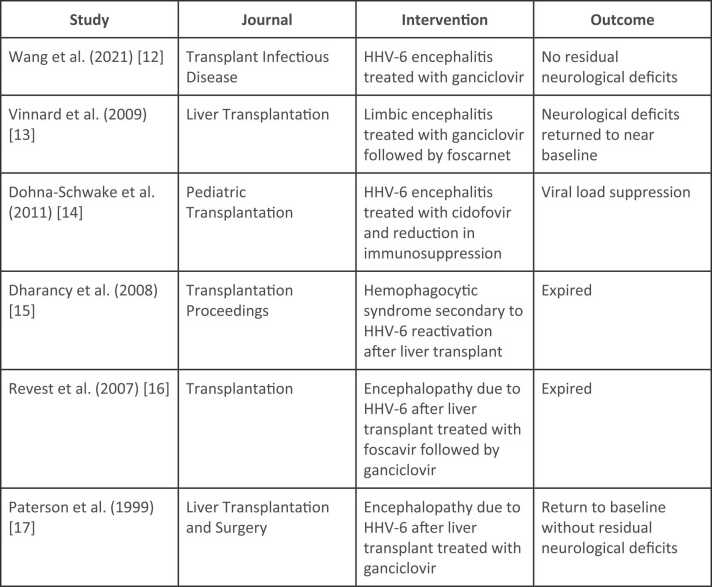
Tabular listing of previous cases of HHV-6 encephalitis in liver transplant patients with associated intervention and outcome.

The clinical manifestations of HHV-6A are elusive, though older reports document an infectious mononucleosis-like syndrome in immunocompetent adults [3]. In the immunocompromised, however, the clinical manifestations of HHV-6A are even more elusive, although unexplained fever, fulminant hepatitis, and encephalitis have been documented in liver transplant recipients as was observed in our patient [6]. HHV-6 is diagnosed by polymerase chain reaction or viral cultures. Laboratory studies in infected patients may show leukocytosis, leukopenia, and/or anemia. The need for imaging is infrequent, though lumbar puncture with cerebrospinal fluid cultures may be performed in the setting of central nervous system involvement as was indicated in our patient [3]. In the immunocompetent patient, HHV-6 is often self-limiting and does not require treatment. In the immunocompromised host, first-line therapy in the treatment of HHV-6 is intravenous ganciclovir until clearance from the blood and cerebrospinal fluid (i.e., approximately three to four weeks). Alternative agents include foscarnet and cidofovir. Reduction in immunosuppressive drugs is also advised. Early antiviral treatment, especially in cases of HHV-6-associated encephalitis, is recommended [3], [12], [13], [14], [15], [16], [17] (Fig. 4).

Early diagnosis and treatment of HHV-6 viremia is critical in the setting of liver transplantation. In a prospective cohort study of 200 consecutive liver transplant recipients, HHV-6 reactivation post-transplantation was associated with the development of invasive fungal infections, multi-dermatomal or disseminated herpes zoster, and cytomegalovirus infection [7]. In a retrospective study of liver transplant recipients, HHV-6 viremia was associated with decreased graft survival and was an independent predictor of late mortality [8].

Recurrence of HHV-6 viral load and clinical symptoms has been widely reported and is difficult to manage. Rate of recurrence in hematopoietic stem cell transplant has been reported to be as high as 30–50% [9]. Vittayawacharin et al. showed that early treatment with a short course of foscarnet may decrease the rate of recurrence in patients status-post hematopoietic stem cell transplant [10]. More data is needed to confirm the efficacy of this therapy and its ability to be extrapolated to solid organ transplant. Reactivation of HHV-6 in liver transplant recipients has been sparsely studied. Fernández-Ruiz et al. demonstrated, in a randomized clinical trial, that routine monitoring of both HHV-6 and HHV-7 did not affect outcomes in liver transplant recipients. This study showed a 35.9% HHV-6 reactivation rate, although it is unclear what fraction of these recurrences were clinically significant leading to acute cellular rejection of the transplant organ [11].

## Conclusion

Recognition of HHV-6 disease by internal medicine physicians, appropriate laboratory assessment, and early institution of therapy is important to decrease morbidity and mortality in liver transplant recipients.

## Ethical approval

Ethics approval was obtained.

## Consent

Informed consent wasobtained from the patient.

## Funding

The authors have no funding sources to declare.

## Credit Authorship Contribution Statement

All authors contributed equally to the study design, writing, and figure generation. The authors were the only contributing parties to this study.

## Declaration of Competing Interest

The authors have no financial or personal disclosures.
